# Exploring the Potential of PEG‐Heparin Hydrogels to Support Long‐Term Ex Vivo Culture of Patient‐Derived Breast Explant Tissues

**DOI:** 10.1002/adhm.202202202

**Published:** 2023-01-04

**Authors:** Maria K. Koch, Akhilandeshwari Ravichandran, Berline Murekatete, Julien Clegg, Mary Teresa Joseph, Madison Hampson, Mitchell Jenkinson, Hannah S. Bauer, Cameron Snell, Cheng Liu, Madeline Gough, Erik W. Thompson, Carsten Werner, Dietmar W. Hutmacher, Larisa M. Haupt, Laura J. Bray

**Affiliations:** ^1^ School of Mechanical Medical and Process Engineering Queensland University of Technology (QUT) Kelvin Grove QLD 4059 Australia; ^2^ Centre for Biomedical Technologies Queensland University of Technology (QUT) Brisbane QLD 4059 Australia; ^3^ Centre for the Personalised Analysis of Cancers Queensland University of Technology (QUT) Translational Research Institute Brisbane QLD 4102 Australia; ^4^ Peter MacCallum Cancer Centre Melbourne VIC 3000 Australia; ^5^ Mater Pathology Mater Hospital Brisbane Mater Health Services Brisbane QLD 4101 Australia; ^6^ Faculty of Medicine The University of Queensland Herston QLD 4006 Australia; ^7^ Cancer Pathology Research Group Mater Research Institute – The University of Queensland Translational Research Institute Brisbane QLD 4102 Australia; ^8^ School of Biomedical Sciences Queensland University of Technology (QUT) Translational Research Institute Brisbane QLD 4102 Australia; ^9^ Leibniz Institute of Polymer Research 01069 Dresden Germany; ^10^ Australian Research Council (ARC) Training Centre for Cell and Tissue Engineering Technologies Queensland University of Technology (QUT) Brisbane QLD 4000 Australia; ^11^ Australian Research Council (ARC) Training Centre for Multiscale 3D Imaging Modelling and Manufacturing (M3D Innovation) Queensland University of Technology (QUT) Brisbane QLD 4000 Australia; ^12^ Max Planck Queensland Center for the Materials Science of Extracellular Matrices Queensland University of Technology (QUT) Brisbane QLD 4000 Australia; ^13^ Centre for Genomics and Personalised Health Genomics Research Centre School of Biomedical Sciences Queensland University of Technology (QUT) Kelvin Grove QLD 4059 Australia

**Keywords:** breast cancer, ex vivo cultures, hydrogels, patient‐derived explants

## Abstract

Breast cancer is a complex, highly heterogenous, and dynamic disease and the leading cause of cancer‐related death in women worldwide. Evaluation of the heterogeneity of breast cancer and its various subtypes is crucial to identify novel treatment strategies that can overcome the limitations of currently available options. Explant cultures of human mammary tissue have been known to provide important insights for the study of breast cancer structure and phenotype as they include the context of the surrounding microenvironment, allowing for the comprehensive exploration of patient heterogeneity. However, the major limitation of currently available techniques remains the short‐term viability of the tissue owing to loss of structural integrity. Here, an ex vivo culture model using star‐shaped poly(ethylene glycol) and maleimide‐functionalized heparin (PEG‐HM) hydrogels to provide structural support to the explant cultures is presented. The mechanical support allows the culture of the human mammary tissue for up to 3 weeks and prevent disintegration of the cellular structures including the epithelium and surrounding stromal tissue. Further, maintenance of epithelial phenotype and hormonal receptors is observed for up to 2 weeks of culture which makes them relevant for testing therapeutic interventions. Through this study, the importance of donor‐to‐donor variability and intra‐patient tissue heterogeneity is reiterated.

## Graphical abstract

Patient‐derived normal breast and breast cancer explant tissues are embedded within poly(ethylene glycol)‐heparin‐maleimide (PEG‐HM) hydrogels and cultured ex vivo for a period of 3 weeks. Evaluating tissue integrity, functionality, and proliferation of embedded versus non‐embedded explant tissues allows for the study and development of alternative methods for long‐term cultures of patient‐derived breast tissues.

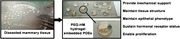

## 1 Introduction

Breast cancer is the second most common cancer worldwide and the leading cancer‐related death in women.^[^ ^1^ ^]^ Current treatment strategies are not sufficient, due to high disease heterogeneity and differences in patient response.^[^ ^2^ ^]^ This necessitates the development of novel treatment strategies, derived from improved knowledge of the disease and its progression.

The tissue microenvironment is crucial for cell behavior and many pathological processes occurring in breast cancers.^[^ ^3^ ^]^ 3D cell culture models overcome limitations of standard 2D culture techniques by better replicating in vivo cell–cell and cell–matrix interactions in breast cancer development.^[^ ^4^ ^]^ However, they cannot fully mimic the complex tissue microenvironment.^[^ ^5^ ^]^ In contrast, animal models, along with the complexity of physiological functionality, require extensive humanization to study the disease in its appropriate microenvironment.^[^ ^6^ ^]^ Patient‐derived explant (PDE) culture models offer an alternative to overcome these limitations. They can replicate key features of the mammary microenvironment with their original tissue integrity and patient cellular heterogeneity and thus better mimic in situ behaviors.^[^ ^7^ ^]^ The commonly used tissue slice culture method has been proven to be viable for 96 h^[^ ^8^ ^]^ and up to 1 week.^[^ ^9^ ^]^ Along with emersion culture, placing the PDE on a substrate such as elevated grids or gel‐like sponges can also help maintain viability for up to 1 week.^[^ ^10^, ^11^, ^12^ ^]^ Moreover, a perfusion‐based bioreactor showed the potential to increase the culture period of explants for up to two weeks.^[^ ^13^ ^]^ However, retaining the viability, tissue integrity, and functionality of PDE cultures over prolonged periods still remains a major challenge. This severely limits the exploration of downstream applications including drug testing and analyses.^[^ ^5^ ^]^

To overcome these limitations, the development of methods for prolonged investigation of PDE cultures would be of immense value. Here, we investigated the use of star‐shaped poly(ethylene glycol) and maleimide‐functionalized heparin (PEG‐HM) hydrogels as semi‐synthetic matrices to support mammary PDEs ex vivo for a prolonged culture time of up to 3 weeks. We compared the culture of unencapsulated PDEs with PDEs encapsulated by PEG‐HM hydrogels under two different media conditions. The morphology of the glandular tissues and stromal tissues were assessed using routine Hematoxylin and Eosin (H&E) staining. The maintenance of tissue functionality was ascertained by staining for epithelial markers and hormonal receptors—estrogen and progesterone. The ex vivo proliferation of the cells was evaluated using staining and quantification for Ki‐67. The project findings have established new methodologies for mammary PDE cultures as a proof‐of‐concept study, providing a biologically relevant platform for future breast cancer research and drug screening.

## 2 Results

### 2.1 Evaluation of Tissue Morphology in Hydrogel‐Embedded versus Control PDE Cultures

Firstly, the pathological analysis of samples was used to define the breast cancer receptor status as estrogen receptor (ER), progesterone receptor (PR), and human epidermal growth factor receptor 2 (HER2) positive (+) or negative (−), as well as the histological subtype of the tissue (**Table** **1**). Table 1 also provides information on which tissues were resected from patients who received neoadjuvant chemotherapy (Donors BC‐4 and NB‐1).

**Table 1:** Overview of patient‐derived normal and breast cancer specimens used in this study. Normal breast tissues (NB, *n* = 3) were received from prophylactic mastectomies from female (f) patients. Breast cancer samples (BC, *n* = 5) were obtained from wide local excisions of tumors (invasive carcinoma or ductal carcinoma in situ, DCIS). Pathological analysis defined the breast cancer tissue as estrogen receptor (ER), progesterone receptor (PR), and human epidermal growth factor receptor 2 (HER2) positive (+) or negative (−). Pathological analysis also defined the histological subtype of the tissue. Some patients had received neoadjuvant chemotherapy prior to tissue collection and processing

Sample type	Donor#	Receptor status	Histological subtype	Donor age	Donor gender	Neoadjuvant chemotherapy
Breast cancer tissue	BC‐1	ER+ PR+ HER2−	No specific type (NST) cribriform carcinoma	33	f	None
	BC‐2	Receptor status unknown (DCIS)	Papillary cribriform carcinoma	41	f	None
	BC‐3	ER− PR− HER2−	Medullary carcinoma	27	f	None
	BC‐4	ER− PR− HER2−	No specific type (NST) carcinoma with a micropapillary component	47	f	Ipilimumab/Nivolumab/Paclitaxel (8 cycles)
	BC‐5	ER+ PR+ HER2−	Pleomorphic lobular carcinoma	64	f	None
Normal breast tissue	NB‐1	N/A	N/A	50	f	Paclitaxel, Trastuzumab for ER/PR/HER2+ lesion (other breast)
	NB‐2	N/A	N/A	44	f	None
	NB‐3	N/A	N/A	36	f	None

**Figure 1:**
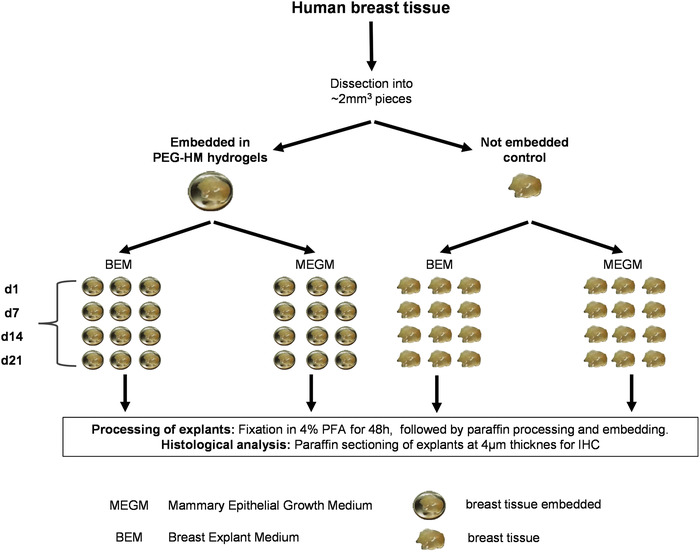
Overview of experimental setup and culture conditions. Received human breast tissue was dissected into pieces measuring ≈2 mm^3^ and embedded into PEG‐HM hydrogels for tissue explant cultures. Non‐embedded tissue served as a control and was cultured in parallel. Explant cultures were maintained for a period of up to three weeks in MEGM or BEM for comparison. For histological analysis, samples were fixed in 4% paraformaldehyde (PFA) on the beginning and after 1 day (d), 7 days, 14 days, and 21 days. After fixation, samples were embedded in paraffin and sectioned.

The obtained tissues varied in size and weight and included glandular as well as adipose tissue. We observed donor‐to‐donor variability in the proportion of adipose tissue to glandular and stroma tissue. The obtained breast cancer tissues were further graded according to the Nottingham grading system for breast cancer (**Table** **2**), which assessed glandular formation, nuclear pleomorphism, and mitosis count of the breast cancer. The explant tissues were manually dissected for culture and therefore varied in size. Then the PDEs were cultured with or without PEG‐HM hydrogel encapsulation for a period of three weeks in two different media conditions denoted as Breast Explant Medium (BEM) and Mammary Epithelial Growth Medium (MEGM). The samples were fixed at different time points for further analyses (**Figure** **1**).

**Table 2:** Overview of histological grading of received breast cancer tissue. Received breast cancer (BC) tissue containing invasive carcinoma was graded by a pathologist, using the Nottingham grading system with a score between 1 and 3, whereby 3 means most abnormal. The table list the features of the tubules grade, nuclear grade, as well as the mitotic rate and its resulting grade. The mitotic rate defines how many dividing cells are present and indicates the growth rate of the tumor. The mitotic rate was based on the assessment of 10 high power fields (hpf), with a field diameter of 0.55 mm

Donor#	Overall histological grade	Tubules grade	Nuclear grade	Mitosis grade (mitotic rate)
BC‐1	2	3	3	1 (7/10 hpf)
BC‐2	N/A	N/A	N/A	N/A
BC‐3	3	3	3	3 (44/10 hpf)
BC‐4	3	3	3	3 (80/10 hpf)
BC‐5	3	3	3	2 (12/10 hpf)

**Figure 2:**
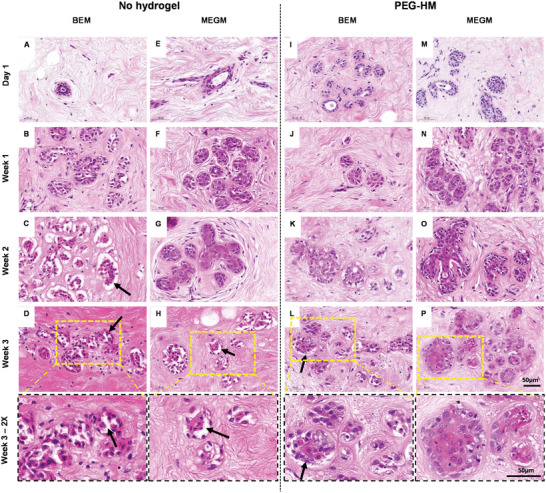
Histology of human mammary glandular tissue of over three weeks of culture. Tissue explant cultures of normal breast tissue were cultured with or without PEG‐HM hydrogel in BEM or MEGM medium. Control tissues were cultured in parallel without prior embedding. For histological examination, explant cultures were fixed at various time points until 21 days and paraffin sections of 4 µm thickness were prepared for H&E staining. Representative brightfield images of epithelial glandular structures including ducts and lobules are shown of NB‐1 explant cultured in A–D) BEM medium without hydrogel embedding, I–L) embedded in hydrogel, E–H) as well as cultured in MEGM medium without hydrogel embedding and M–P) embedded in hydrogel. Yellow dotted line (D, H, L, P) demonstrates area of higher magnification in week 3–2× row. Scale bar: 50 µm.

**Figure 3:**
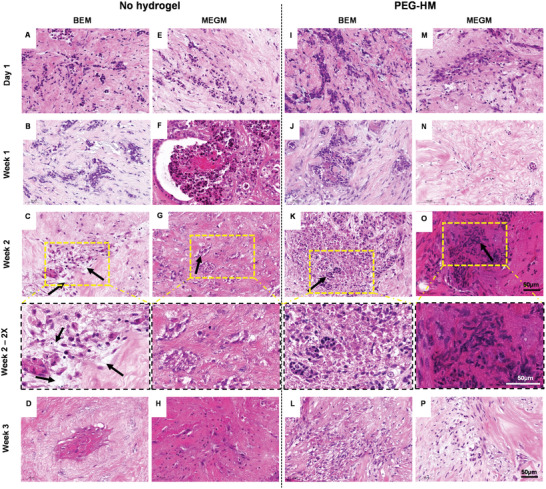
Histology of human breast cancer tissue over three weeks of culture. Tissue explant cultures of breast cancer tissues were cultured embedded with or without PEG‐HM hydrogel in BEM or MEGM medium. Control tissues were cultured in parallel without prior embedding. For histological examination, explant cultures were fixed at various time points until 21 days and paraffin sections of 4 µm thickness were prepared for H&E staining. Representative brightfield images of epithelial structures are shown of BC‐1 explant cultured in A–D) BEM medium without hydrogel embedding, I–L) embedded in hydrogel, E–H) as well as cultured in MEGM medium without hydrogel embedding and M–P) embedded in hydrogel. Yellow dotted line (C, G, K, O) demonstrates area of higher magnification in week 2–2× row. Scale bar: 50 µm.

Firstly, the ex vivo mammary explant cultures derived from normal breast (NB) tissues and breast cancer (BC) tissues were sectioned and stained forH&E to evaluate tissue integrity and architecture. Representative images of H&E‐stained tissue sections show the glandular tissues for both the NB (**Figure** **2**) and BC tissues (**Figure** **3**) on day 1, weeks 1, 2, and 3 of culture in either BEM or MEGM medium.

In the normal breast tissues, the overall glandular architecture was seen to be preserved throughout all culture conditions for up to two weeks. However, cellular changes could be observed in the epithelium of the glandular structures. At 3 weeks, some epithelial cells became dissociated from the basement membrane (Figure 2D, H, L; arrows) compared to the earlier timepoints of culture (Figure 2A, E, I, M). Cells also exhibited nuclear shrinkage and loss of cytoplasm, morphologic features suggestive of apoptosis. This effect was more dominant in explants cultured in BEM without hydrogel embedding. In this culture condition, the described effect was observed after two weeks of culture (Figure 2C; arrow) in comparison to the other conditions at the same timepoint (Figure 2G, K, O). The stromal tissue (Figure S1, Supporting Information) surrounding the glandular tissue in the mammary explant cultures were mostly made of connective tissue (pink) with relatively low cellularity and a small proportion of adipose cells (white globules). This was due to the processing of the explants where most of the adipose tissue was removed prior to culture. H&E‐stained connective tissue showed a woven structure of fibers with few adipose cells. The degree of woven to more aligned fibers differed throughout the tissue and resulted in heterogeneity between the tissue pieces. The difference in the intensity of the (predominantly eosin) staining showed the variation of the stromal tissue density between the different PDE samples of the same donor throughout the tissue. No predominant difference between the various culture conditions could be observed in the stromal tissue over time.

In the case of BC, we observed heterogeneity in the distribution of glandular structures across the different donors and replicates studied (Figure 3). As expected, the neoplastic epithelial cells were infiltrative and disorganized, and the tumor showed a diffuse solid architecture (Figure 3A, E, I, M). By the second week of culture, we observe formation of pockets within the cells in the non‐embedded BEM cultures (Figure 3C, black arrow) when compared to the other groups at the same time point (Figure 3G, K, O, black arrow). Apoptotic and necrotic nests of cells in un‐embedded control PDEs showed evidence of retraction in the tissue giving way to clear spaces around nests of tumor cells. The breast cancer PDEs showed a more dense stroma in comparison with normal breast tissue, especially when embedded in PEG‐HM hydrogels (Figure S2, Supporting Information). This denser stromal tissue, detected by increased eosinophilia on H&E staining, was still visible after three weeks of culture in MEGM (Figure S2P, Supporting Information).

The H&E images of sections from all the donors were graded by a pathologist (**Table** **3**) using the following scoring system: Score 5—Pristine, Score 4—Minor nuclear and cytoplasmic changes (enlargement and vesiculation), Score 3—Easily identifiable nuclear and cytoplasmic changes, Score 2—Significant degenerative changes (pyknosis, cytoplasmic blurring), Score 1—Complete loss of cellular detail (loss of nuclei and abundant debris). The PEG‐HM hydrogels, especially in combination with MEGM appeared to have a protective effect around day 7 of culture. At day 14 and day 21, PEG‐HM hydrogels in combination with BEM offered the improved preservation of normal breast tissue features and some improved preservation of breast cancer tissue features, although was highly dependent on the donor tissue.

**Table 3:** Scoring of the tissue preservation and structural integrity of explant cultures. H&E images of the normal breast tissues and breast cancer explant tissues cultured with and without hydrogel embedding over a period of 3 weeks were scored by a pathologist using the following scoring system: Score 5—Pristine; Score 4—Minor nuclear and cytoplasmic changes (enlargement and vesiculation); Score 3—Easily identifiable nuclear and cytoplasmic changes; Score 2—Significant degenerative changes (pyknosis, cytoplasmic blurring); Score 1—Complete loss of cellular detail (loss of nuclei and abundant debris)

Breast explant samples		Normal breast			Breast cancer				
		NB‐1	NB‐2	NB‐3	BC‐1	BC‐2	BC‐3	BC‐4	BC‐5
Day 1	d1_noGel‐BEM	2	4	3	2	2	2	2	2
	d1_noGel‐MEGM	2	3	3	2	2	2	1	2
	d1_PEG‐BEM	3	3	3	2	3	1	1	1
	d1_PEG‐MEGM	2	3	4	1	3	1	1	1
Day 7	d7_noGel‐BEM	2	2	3	1	3	2	2	2
	d7_noGel‐MEGM	2	2	3	2	3	2	1	2
	d7_PEG‐BEM	3	3	3	1	3	2	1	2
	d7_PEG‐MEGM	3	2	3	1	3	3	1	3
Day 14	d14_noGel‐BEM	2	2	2	1	3	3	2	2
	d14_noGel‐MEGM	2	2	3	1	2	2	1	3
	d14_PEG‐BEM	3	3	3	2	3	1	2	3
	d14_PEG‐MEGM	3	2	3	1	2	2	1	2
Day 21	d21_noGel‐BEM	2	2	2	1	2	3	1	3
	d21_noGel‐MEGM	2	2	3	1	2	2	1	3
	d21_PEG‐BEM	2	3	3	2	3	3	1	3
	d21_PEG‐MEGM	2	2	3	1	2	2	1	3

In addition to tissue morphology, the H&E images were also examined to assess the integrity of the PDE periphery over time between different conditions and donors (Figure S3, Supporting Information). The compactness of the PDE is donor‐ and tissue‐dependent and seems to be defined at the start of the culture (Figure S3A, Supporting Information). The donor tissue with a compact surface structure (Figure S3A, Supporting Information) stayed stable over the three week culture period, with or without PEG‐HM hydrogel support (Figure S3B,C, Supporting Information). If a PDE at the start of culture showed a less defined periphery with more loose surface structures and separating tissue fibres (Figure S3D; arrow, Supporting Information), the same can be seen with the non‐embedded controls by the end of 3 weeks (Figure S3E; arrow, Supporting Information). However, PDEs embedded in PEG‐HM hydrogels showed a smooth tissue periphery without ruptures at the interface of the tissue to the surrounding hydrogel (Figure S3F; arrow, Supporting Information) similar to compact donor tissue (Figure S3A–C, Supporting Information) showing the mechanical support of the PEG‐HM hydrogel.

### 2.2 Maintenance of Tissue Functionality in Hydrogel‐Embedded versus Control PDE Cultures

**Figure 4:**
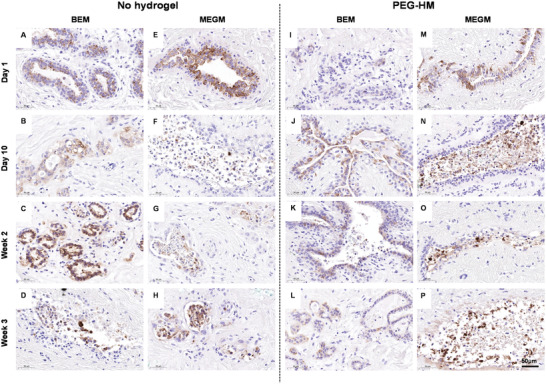
Epithelial staining of normal breast (NB‐2) explant cultures. Tissue explant cultures of normal breast tissue were cultured embedded in PEG‐HM in BEM or MEGM medium. Control tissues were cultured in parallel without prior embedding. To confirm the presence of epithelial cells in the mammary tissues, 4 µm thick sections of explant cultures were stained for Cytokeratin 8/18. Representative brightfield images of epithelial glandular structures are shown of NB‐2 explants cultured in A–D) BEM medium without hydrogel embedding, I–L) embedded in hydrogel, E–H) as well as cultured in MEGM medium without hydrogel embedding and M–P) embedded in hydrogel. Scale bar: 50 µm.

**Figure 5:**
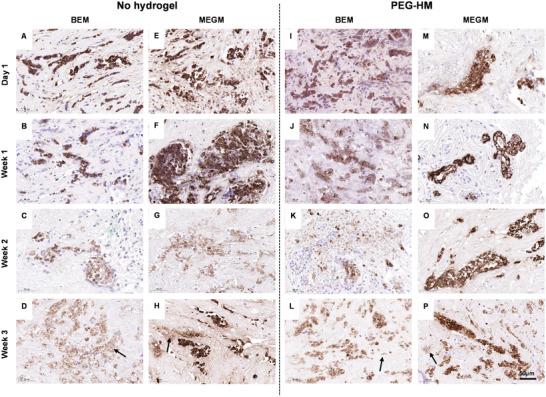
Epithelial staining of breast cancer explant cultures. Tissue explant cultures of breast cancer tissues were cultured embedded inPEG‐HM in BEM or MEGM medium. Control tissues were cultured in parallel without prior embedding. To confirm the presence of epithelial cells in the mammary tissues, 4 µm thick sections of explant cultures were stained for Cytokeratin 8/18. Representative brightfield images of epithelial glandular structures are shown of BC‐1 explants cultured in A–D) BEM medium without hydrogel embedding, I–L) embedded in hydrogel, E–H) as well as cultured in MEGM medium without hydrogel embedding and M–P) embedded in hydrogel. Scale bar: 50 µm.

The ex vivo culture of patient derived explant tissues for tumor modelling and drug testing necessitates the maintenance of tissue functionality for the duration for culture. Specifically for breast cancer diagnosis and targeting, the presence of hormonal receptors is crucial for hormone receptor‐based therapeutic testing. Firstly, we confirmed the epithelial nature of the glandular tissues in both the normal breast (**Figure** **4**, Figure S4, Supporting Information) and breast cancer explants (**Figure** **5**, Figure S5, Supporting Information) by staining for epithelial marker, cytokeratin 8/18 (CK8/18). Irrespective of condition, patients with stronger epithelial staining on Day 1 (e.g., NB‐2, Figure 4 and BC‐1, Figure 5) continued to retain that for the remaining period of culture. Even in these tissues, we observe the CK8/18 staining to be more diffuse by the third week of culture (Figure 5D, H, L, P; marked by arrows) coinciding with loss of integrity in the epithelium and tissue periphery.

**Figure 6:**
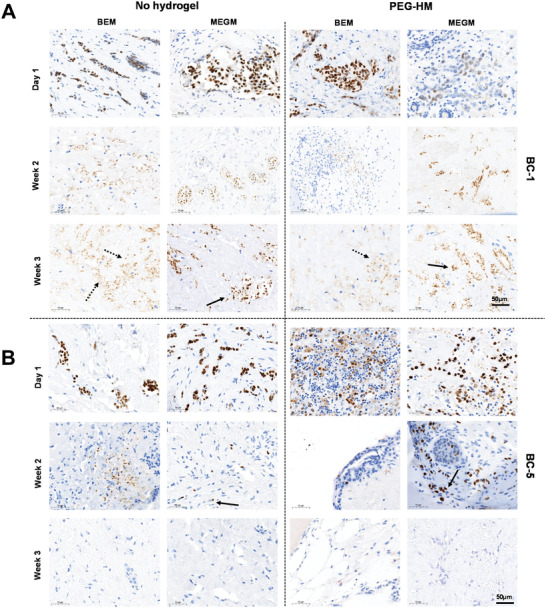
Immunohistochemical staining for ER in BC‐1 and BC‐5. Tissue explant cultures of breast cancer tissues were cultured embedded in PEG‐HM in BEM or MEGM medium. Control tissues were cultured in parallel without prior embedding. Representative brightfield images of immunohistochemistry (IHC) staining for estrogen receptor are shown for day 1, week 2, and week 3 samples of A) BC‐1 and B) BC‐5 explants cultured in BEM/MEGM medium with and without hydrogel embedding. Scale bar: 50 µm.

**Figure 7:**
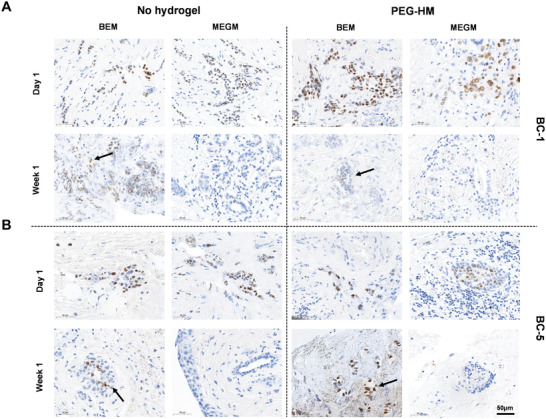
Immunohistochemical staining for PR in BC‐1 and BC‐5. Tissue explant cultures of breast cancer tissues were cultured embedded in PEG‐HM in BEM or MEGM medium. Control tissues were cultured in parallel without prior embedding. Representative brightfield images of IHC staining for progesterone receptor are shown for day 1 and week 1 samples of A) BC‐1 and B) BC‐5 explants cultured in BEM/MEGM medium with and without hydrogel embedding. Scale bar: 50 µm.

Next, we tested the presence and maintenance of hormonal receptors ER and PR for breast cancer patients BC1 and BC5 (ER+/PR+) over the 3‐week culture period. Scans of ER and PR staining from the original pathology analysis of the tissue were used as a baseline to compare it with the receptor status of the ex vivo cultured explant tissues over time (Figure S6, Supporting Information). ER expression was maintained in the explant tissues for more than 2 weeks of culture (**Figure** **6**). Although, the staining tends to become more diffuse with time (Figure 6; dotted arrows), strong positive staining was observed in the MEGM samples in week 3 as well (Figure 6A; solid arrow). Interestingly, the ER status seemed to be more effectively maintained in MEGM media conditions when compared to the BEM media conditions in both the hydrogel embedded and control PDEs. On the other hand, the explants maintained the presence of PR until the first week of culture (**Figure** **7**) and there was no positive staining for PR in the tissues in the week 2 and week 3 sections from both the breast cancer patients BC1 and BC5 (ER+/PR+) across all groups (data not shown). At week 1, culturing the tissues in BEM media condition seemed to have a positive effect in helping retain the progesterone receptors (Figure 7A, B; arrows).

### 2.3 Proliferation in PDE Breast Cultures

**Figure 8:**
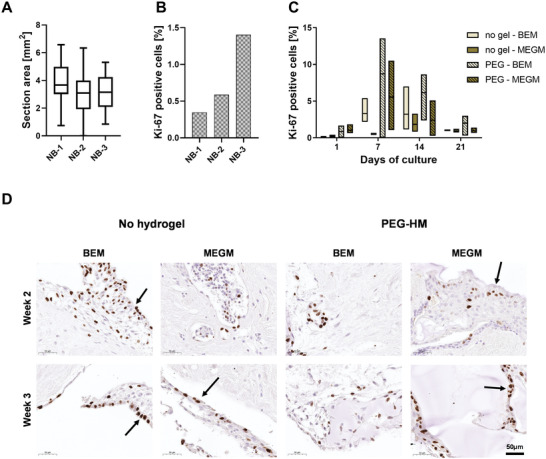
Proliferation in normal human breast explant cultures. Proliferation in human breast explants at various time points and culture conditions was determined immunohistochemically by staining for Ki‐67. Positive cells were quantified for the whole explant region of triplicates of each culture condition and differences can be seen between the various time points and culture conditions. A) The size variation of the sections per donor (*n* = 46–51) in mm^2^. Boxplot whiskers are plotted by Tukey and the line shows the median. B) Percentage of positive cells for Ki‐67 of non‐cultured tissue (*n* = 3) at day 0 shown for all three normal breast donors. C) Quantification of Ki‐67 positive cells of donor NB‐1 tissue explants showing the proliferative activity over time from day 1 to day 21 and at various culture conditions. Box plot represents the three replicates for each tissue with line at median. D) Representative brightfield images of Ki‐67‐stained sections of NB‐1 are shown for week 2 and week 3. Sections were counterstained with hematoxylin. Scale bar: 50 µm.

Next, we evaluated the proliferative activity of the breast cancer tissue using the proliferation marker Ki‐67 to determine if the different culture conditions affected the proliferation of the mammary cells. The whole tissue section was used for Ki‐67 quantification and measurement of the area showed that most sections were between 3 and 4 mm^2^ (**Figure** **8**A). Normal breast tissue was collected directly after the surgery, which aided to minimize the time between surgery and the processing of the tissue in the laboratory. To determine the activity of the PDEs before culture, samples were fixed, and Ki‐67 was quantified after tissue processing was completed but before culture started (non‐cultured d0) (Figure 8B). The proliferative activity of triplicates of donor NB‐1 and NB‐2 range between 0.4% and 0.6%, while NB‐3 showed an activity of 1.4% (Figure 8B). As an example, Figure 8C shows the percentage of Ki‐67 positive cells to the total count of cells per culture condition of NB‐1. A general increase in proliferation within a week could be observed followed by a decrease to almost the same level than in the beginning after three weeks in culture (Figure 8C). The quantification of NB‐1 showed, that throughout the culture period, PDEs embedded in PEG‐HM hydrogels showed more proliferative cells in comparison to the corresponding controls (non‐embedded explants). Culturing the PDEs in BEM medium in comparison to MEGM seemed to further improve the proliferative activity in this tissue after 7, 14, and 21 days in culture. Looking at the non‐embedded controls only, the BEM medium showed to have a supportive effect in explants up to two weeks in culture. Representative brightfield images of the Ki‐67 staining of NB‐1 at the end of week 2 (Figure 8D) and week 3 of culture (Figure 8D) demonstrate this decrease of proliferative activity. Also, the images show the tendency of proliferating cells to be located at the periphery of the tissue in all the groups, which is in closest contact with the culture medium (Figure 8D; arrows). The other two cultures of different donor confirmed the observed increase in proliferation within a week of culture followed by a decrease towards 3‐weeks (Figure S7, Supporting Information). It should also be noted that the levels and pattern of proliferative activity reasonably vary between donors, although embedding the PDEs seemed to have a positive effect for proliferation (Figure S7, Supporting Information). Overall, proliferative activity in normal breast tissue were detected even after three weeks in culture in a similar or even higher amount compared to the tissue before culture.

**Figure 9:**
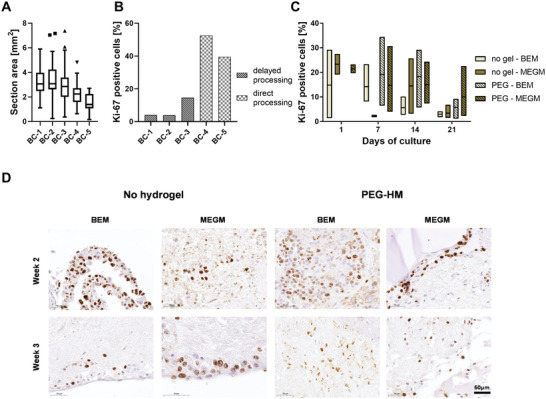
Proliferation in human breast cancer explant cultures. Proliferation in human breast cancer explants at various time points and culture conditions was determined immunohistochemically by staining for Ki‐67. Positive cells were quantified for the whole explant region of triplicates of each culture condition and differences can be seen between the various time points and culture conditions. A) The size variation of the sections per donor (*n* = 41–51) in mm^2^. Boxplot whiskers are plotted by Tukey and the line shows the median. Outliers are marked as symbol above and below the whiskers. B) Percentage of positive cells for Ki‐67 of non‐cultured tissue at day 0 shown for all five breast cancer donors. Donors were categorized into direct processing, where the tissue was collected on the same day of surgery after the pathological assessment of the tissue. Tissue collected after an overnight storage of the tissue in the fridge is labeled as delayed processing. C) Quantification of Ki‐67 positive cells of donor BC‐4 tissue explants showing the proliferative activity over time from day 1 to day 21 and at various culture conditions. Box plot represents the three replicates for each tissue with line at median. D) Representative brightfield images of Ki‐67‐stained sections of BC‐4 are shown for week 2 and week 3. Sectioned were counterstained with hematoxylin. Scale bar: 50 µm.

The majority of the sections from breast cancer tissues averaged between 1 and 4 mm^2^ in area (**Figure** **9**A). In the case of breast cancer tissues, there was a variability between the tissues in terms of time taken between surgery and tissue processing. There was a time delay between surgery and tissue processing for BC‐1, BC‐2, and BC‐3 whereas the remaining two tissues (BC‐4 and BC‐5) were collected and processed right after surgery. Consequently, the quantification of the Ki‐67 positive cells in tissue explants (Figure 9B) showed the difference in the level of proliferative cells present in BC‐1, BC‐2, and BC‐3 versus BC‐4 and BC‐5. This difference in proliferative activity was also clearly visible in brightfield images of the day 0 stained tissue sections (data not shown). In general, the breast cancer tissue explants showed higher proliferative activity than the normal breast tissues, which was expected. As an example, the proliferative activity of the ER‐ PR‐ HER2‐ tumor of donor BC‐4 is presented in Figure 9C where we observe a general decline after 14 days of culture. Throughout culture, explants embedded in PEG‐HM hydrogels showed a higher proliferation of cells in comparison to non‐embedded controls, with the exception of controls cultured in MEGM medium. The difference was evident in the representative images of Ki‐67 staining at day 14 and day 21 (Figure 9D). A high variation could be detected between different breast cancer tissue donors and consecutive different breast cancer types (Figure S8, Supporting Information). For instance, the overall proliferative activity in BC‐2 tissue at the beginning of the culture is at the same range as the week 3 tissues from donor BC‐4 (Figure S6, Supporting Information). Besides the exception of non‐embedded controls cultured in BEM at week 1, PEG‐HM embedded explants showed a slightly higher trend of proliferative activity amongst the different groups, especially after 14 days in culture.

## 3 Discussion

PDE cultures have gained a lot of attention in the past couple of years owing to their ability to recapitulate tissue structure and concomitant function in more complexity than artificial in vitro models. It has been demonstrated that the interaction of epithelial and stromal tissues is important for epithelial maintenance and plays an important role in malignant transition to cancer.^[^ ^14^, ^15^ ^]^ The advantage of tumor PDEs are that cells from both the tumor and stroma in the explants can connect and communicate ex vivo via inter‐ and extracellular pathways.^[^ ^16^ ^]^

Patient‐derived breast tissue explants have been cultured ex vivo predominantly as organotypic tissue slice models for a period of 2 days up to 3 weeks. In the era of personalized medicine, these explant cultures could enable the drug selection by assessment of effects on breast tissues over time. However, for these drug testing studies, it becomes very important to maintain tissue architecture in the mammary tissue explants beyond a few days. Thin precision‐cut slices of tissue have been used to determine the effect of drugs on the tissue and its toxicity.^[^ ^8^, ^17^, ^18^, ^19^ ^]^ The disadvantage of this approach is that tissue slices do not last longer than a week before disintegrating, which allows only a short treatment and observation time. To overcome this issue, several studies have demonstrated the use of a commercially available gelatine sponge as a substrate to support the explant slice and help prevent cellular outgrowth.^[^ ^20^ ^]^ However, it is important to note that tissue slices have an optimal infiltration of medium and the treatment components,^[^ ^21^ ^]^ which does not accurately mimic in situ situations. The complexity and size of the tissue or the tumor in breast tissue prevents complete infiltration of nutrients or a drug, which leads to reduced sensitivity to treatment^[^ ^22^ ^]^ and a necrotic core of the tumor upon reaching a certain size.^[^ ^23^ ^]^ Extrapolating the observations of organotypic slice cultures from the literature and comparing it with the current study, we observe that a supportive scaffold aids in maintaining tissue integrity and architecture better than free floating/direct well plate cultures. Here, we used PEG‐HM hydrogels with adequate mechanical properties to provide support to the explant tissue for longer durations without loss of hydrogel structural integrity. We showed that human mammary PDEs from normal as well as breast cancers can be maintained as viable tissues with intact tissue architecture for a culture period of 2–3 weeks

Histological analyses of the cultured tissue revealed a supportive effect of the PEG‐HM hydrogels when compared to the non‐embedded controls. Further, the results indicated that the supportive structure of the hydrogel held more benefit for maintaining tissue architecture than a more specialized nutrient cocktail. Beyond qualitative assessments described in the results, we reported a new scoring system in this study to quantify the maintenance of tissue structure and integrity from H&E images. However, we realize that the H&E based scoring for patient derived tissues can be complicated because it may be less relevant to have a uniform scoring for cancers that present very different characteristics. For instance, if one of the cancers is a classic invasive lobular carcinoma and another is a high‐grade invasive carcinoma of no special type, they have different interpretation issues. With the lobular carcinoma, the cells are small and rather uniform naturally, and the morphology changes are difficult to appreciate. With the high‐grade carcinoma, the tumor may show extensive necrosis and cell loss natively, which would be interpreted as “complete loss of cellular detail” (i.e., Score 1). However, this could be an endogenous tumor factor rather than due to the gel or lack thereof.

Another important challenge with PDEs is the intra‐sample tissue heterogeneity leading to difficulties in reporting quantifiable effects. Due to the relatively large tissue explants (2–3 mm^3^) used in our study, the histopathological assessment involved random sampling of tissue sections. This makes the assessment of intra‐tumoral heterogeneity challenging. We cannot assume the presence of glandular epithelial structures in the replicates of an explant microtissue, which may be necessary for further analyses. Alternatively, the tissues that are not representative can be eliminated from the further downstream analyses that may result in variable tissue numbers across different groups.^[^ ^24^ ^]^ For instance, in the CK8/18 staining of the BC‐5 samples in our study, it is hard to gauge if the chosen tissue sections/microtissues themselves were more stromal than glandular or if it was representative of the patient's inherent tissue characteristics. Kokkinos et al. addressed this intra‐tumoral heterogeneity by using explants from three distinct regions of pancreatic tumor tissue for each group of study.^[^ ^25^ ^]^ And Centenera et al. performed routine H&E on day 0 to identify the presence of tumor cells before using the tissues for further analyses.^[^ ^20^ ^]^ Methods to account for this heterogeneity should be further explored in future breast explant studies, where a greater number of patients per subtype would need to be studied before making any inferences.

One of the main therapeutic strategies in ER+/PR+ breast cancers involve targeting the hormonal receptor signaling pathways as they have been known to be crucial for carcinogenesis. Additionally, disease progression in over 30% of these cases is attributed to resistance to hormonal therapy.^[^ ^26^ ^]^ This highlights the importance of maintenance of hormonal receptors in the explants for a considerable duration (>3 days) to identify new targets and study patient‐dependent resistance mechanisms.^[^ ^26^ ^]^ In our study, we showed the presence of ER and PR markers in ER+/PR+ PDEs (BC‐1 and BC‐5) for 2 weeks and 1 week respectively. Our results revealed the importance of role of choosing relevant medium conditions for prolonged hormonal receptor activity.

In our study, we used Ki‐67 as a proliferation marker which is expressed throughout the active phases of the cell cycle.^[^ ^27^ ^]^ Our comparison of normal to breast cancer tissue showed a clear difference in the higher proliferation potential of malignant cells, a hallmark of cancer.^[^ ^28^ ^]^ Normal breast tissue only included a small proportion of proliferative active cells, and the state of activity was shown to be dependent on various factors, such as the menstrual cycle or age.^[^ ^29^ ^]^ These variations could be observed between various donors, enabling the use of patient‐derived tissue to replicate patient heterogeneity in terms of age, parity, and maturity of the organ. Comparing the proliferative activity of embedded PDEs in PEG‐HM hydrogels to non‐embedded controls cultured in BEM or MEGM, we could not draw conclusive results regarding the optimal culture condition, due to the high variation between the groups, donors, and breast cancer types and this may require further assessment. In general, an increase of proliferation was measured in the first week of culture, showing the proliferative support of the high nutrient supply of in vitro cultures. Due to a gradient of nutrients into the PDE using the presented explant culture method, more proliferative cells were detected closer to the surface than in the core, which mimics more closely the in situ tumor. While comparing PDEs to the original tumor, it is important to recognize the role of media and note the differences in proliferation between uncultured and cultured tissues. These are signs of adaption to in vitro conditions which can potentially change characteristics of the tumor.

Another critical factor in explant culture, shown by the quantification of proliferative cells is the time between collection and culture initiation. Due to availability of the donated tissue for research purpose, BC‐4 and BC‐5 could be collected after pathological assessment of the tissue at the hospital, while BC‐1, BC‐2, and BC‐3 could only be collected the next morning and were kept in medium at 4 °C. Comparing the Ki‐67 quantification of received breast cancer tissues to the pathological assessment at the hospital, a reducing effect on proliferative activity could be observed between two donors with a similar breast cancer type, underlining the importance of a shorter time between tissue collection and processing. In addition to the delay in processing times, the differences between breast cancer donors in terms of the subtypes (Table 1) and cancer grades (Table 2) should also be taken into consideration while reporting the data. While BC‐1 (ER+ PR+ HER2‐) was processed delayed, it was also histologically graded as 2 with a mitotic grade of only 1, representing a low number of proliferative cells present. Although the mitotic grade of BC‐2 was not determined by the pathologist, the tumor was defined as non‐invasive DCIS and showed a similar proliferative activity as BC‐1 after the same treatment and processing time. In contrast, BC‐3 as an ER‐ PR‐ HER2‐ defined cancer showed higher proliferation then BC‐1 and BC‐2, although they were processed similarly. However, the difference between BC‐4 and BC‐5 was interesting. Although BC‐4 was also mitotic grade 3, the mitotic count was almost doubled (80/10 hpf) in comparison to BC‐3 (44/10 hpf). Nevertheless, the proliferative level was lower than of BC‐5 (ER+ PR+ HER2−) at the start of the PDEs culture and the mitotic grade of BC‐5 was 2 with a mitotic count of 12/10 hpf. The quantification of the Ki‐67 positive cells shown in comparison to the pathological assessment of the tumor showed the importance of consideration of the heterogeneity between breast cancer types and patients.

Additionally, it is important to note that the effects observed above could also be a consequence of the neoadjuvant chemotherapy administered to some of the patients prior to tissue collection for this study. Out of the breast cancer samples, BC‐4 received neoadjuvant chemotherapy (Ipilimumab/Novolumab/Paclitaxel weekly: 8 cycles) before the tumors were resected. Amongst the normal breast tissues, NB‐1 was processed after the patient received neoadjuvant chemotherapy (Paclitaxel/Trastuzumab) for a malignancy in their opposite breast. This may explain why the tissues obtained from these two donors both scored lowest for tissue preservation and structural integrity (Table 2) at the beginning of data collection (day 1) for malignant and normal tissues respectively.

In the presented ex vivo culture model, the integrity of the architectural structure of breast tissue, including the changes in the stroma and the epithelium, can be maintained over three weeks of culture. Differences in the compactness of tissue between donors, which could lead to an earlier disintegration was shown to be prevented by embedding the tissue in PEG‐HM hydrogels. The support kept the tissue compact, like surrounding tissue in the body, while at the same time, enabled infiltration with nutrients shown by an increase in proliferation in culture. This can be seen by the maintenance of actively proliferating cells in normal breast tissue up to three weeks in our model, indicating a stable tissue culture. The decision of maintaining patient‐derived tissue ex vivo and extending the culture time of PDEs potentially up to 3 weeks should be made in accordance with the research question. It is important to note that while histological changes in tissue preservation and hormone receptor status were compared in this study, experiments phenotypically comparing freshly resected tumor tissue with the encapsulated samples are missing and should be considered for future experiments. Nevertheless, this proof‐of‐concept model allows for the study of the interactions between epithelial and stromal tissue in a tissue‐relevant microenvironment for future studies to gain more insight into breast cancer development and treatment response in vitro. The intact structure of breast tissue combined with maintenance of functional epithelium provides a good platform for use in future breast cancer research and personalized medicine applications, however the clinical relevance of the model remains to be shown. With this model, future studies could explore longitudinal evaluation of tissue phenotype and response to pharmacological testing. For example, the use of tissue slices and a larger patient cohort for each breast cancer subtype combined with state‐of‐the‐art RNA sequencing and profiling techniques can be used to evaluate similarities to the original freshly resected tissue and its response to therapeutic interventions.

## 4 Conclusion

In this study, we present an ex vivo PDE model using supportive PEG‐HM hydrogels to culture patient‐derived normal and breast cancer tissue for an extended period. The data showed the culture of the PDEs with stable tissue architecture and functionality up to 2–3 weeks, allowing long‐term observations of the mammary tissue or tumor in its in situ‐like architecture. The results underlined the heterogeneity of the tissue between cancer types, patients, and challenges in evaluating and comparing them. Further, the study demonstrated the effect of choice of culture medium on sustenance of hormone receptors in the explant cultures. Also, the heterogeneity across samples revealed that the culture conditions should be further examined and adapted to improve optimal maintenance of the PDEs ex vivo.

## 5 Experimental Section

### Human Ex Vivo Mammary Tissue

PDEs derived from human breast tissue were obtained from prophylactic mastectomies for normal breast tissue (NB) (*n* = 3) and wide local excisions of breast cancer (BC) (*n* = 5) following informed consent. Ethics for the use of non‐pathological and breast cancer tissue was approved by the Metro South, Mater and QUT Human Research Ethics Committees (ethics approval numbers HREC/16/QPAH/107, HREC/17/MHS/50, and QUT ethics 1700000816). The breast tissues derived from female patients at the ages of 33–64 years were examined by a pathologist prior to donation for research. The samples were transported to the laboratory on ice in RPMI‐1640 including 10% (v/v) fetal bovine serum (FBS; both Gibco, ThermoFisher Scientific, Scoresby, VIC, Australia) and 1% (v/v) Antibiotic‐Antimycotic solution (Anti‐Anti; Sigma‐Aldrich, Castle Hill, NSW, Australia).

### Explant Culture Preparation and Maintenance

Synthetic four‐armed star‐shaped poly(ethylene glycol) (PEG) functionalized with matrix metalloprotease (MMP) cleavable peptide sequences and maleimide‐functionalized heparin (HM) components were synthesized as described previously.^[^ ^30^ ^]^ PEG‐HM hydrogels, formed by Michael‐type addition from the components were prepared as described previously.^[^ ^31^ ^]^ The molar ratio of PEG to heparin‐maleimide was set at 𝜸 = 1 to obtain a stiffness of ≈1.5 kPa (storage modulus) for explant tissue embedding.^[^ ^31^ ^]^ Briefly, the starPEG‐peptide and heparin‐maleimide components were dissolved in phosphate‐buffered saline (PBS; Gibco, ThermoFisher Scientific, Scoresby, VIC, Australia). For embedding, 20.8 mg of PEG and 20.2 mg of HM were each dissolved in 450 µL PBS to generate a volume of 900 µL of PEG‐HM hydrogel material for each donor tissue. Received human mammary tissue was handled under sterile conditions and soaked in PBS. First, glandular tissue was separated from adipose tissue as much as possible and only glandular tissue was used for explant cultures. The explant tissue was then dissected into small pieces of ≈2–3 mm^3^ in size using a scalpel. Tissue pieces were placed on a slide coated with Sigmacote (Sigma) at least 1 cm apart. The dissolved PEG and HM solutions were mixed at a 1:1 ratio and pipetted on top of an explant to create a 30 µL droplet. In situ cross‐linking of the hydrogel occurred within 3 min and could be altered by changing the pH if necessary. Control samples were cultured under the same conditions without prior hydrogel embedding.

The mammary explants were then transferred to 24‐well plates (Thermo Scientific) and covered with 1.5 mL of Mammary Epithelial Growth Medium (MEGM, Lonza, Mount Waverly, VIC, Australia) or breast explant medium (BEM), comprised of RPMI‐1640 (Life Technologies) supplemented with 10% fetal bovine serum (Gibco), 1% antibiotic/antimycotic (Sigma), 10 µg mL^−1^ hydrocortisone (Sigma), 10 µg mL^−1^ human insulin (Sigma), and 20 ng mL^−1^ human epidermal growth factor (Sigma). PDEs were cultured in triplicates for up to three weeks at 37 °C and 5% CO_2._ During culture, 1 mL^−1^ of the medium was changed twice per week by gentle removal and replacement of the medium until samples were fixed at the respective time points. One set of samples were fixed immediately after hydrogel embedding as non‐cultured controls.

### Histology

For histological analysis, non‐cultured (day 0) and cultured PDEs were fixed in 4% paraformaldehyde (PFA) for 48 h at 4 °C. Samples were then dehydrated and processed in an automated Excelsior ES tissue processor with a processing time of ≈5 h (ThermoFisher Scientific, Waltham, USA) before embedding in paraffin. Triplicate or duplicate samples from the same condition were embedded in the same paraffin block. For staining, consecutive paraffin sections of 4 µm were cut from each block using the microtome (Leica RM2265). Sections of each sample from various donors were then stained with hematoxylin and eosin (H&E) using the XL High Throughput Autostainer (Leica) and the Robotic Coverslipper (Leica). The stained slides were imaged at 40× magnification using a slide scanner (3D Histech).

### Immunohistochemistry

The histological assessment of the tissue included immunohistochemistry (IHC) staining against the epithelial marker CK8/18, proliferation marker Ki‐67, Estrogen Receptor (ER) and Progesterone receptor (PR). The IHC staining for CK8/18 (Novus Bio, clone K8.8+DC10, 1:100), and Ki‐67 (Dako, clone MIB‐1, 1:100) was performed manually. Briefly, paraffin sections were dewaxed in xylene and rehydrated through a graded alcohol series followed by demineralized water. Heat‐based antigen retrieval was performed either using by Tris‐EDTA buffer (pH 9) or Sodium Citrate buffer (pH 6) in a decloaking chamber. This was followed by blocking the endogenous peroxidase by incubating in a solution of 3% Hydrogen Peroxide (H_2_O_2_) in PBS for 5 min before incubation with 2% Bovine Serum Albumin in PBS for 30 min at RT to block non‐specific binding. Samples were then incubated with primary antibody diluted in the blocking buffer 1:100. Human tonsil served as a positive control tissue. Each slide contained a stained section and a non‐stained section serving as a negative control. Signal detection was performed using Envision Dual link system‐HRP from DAKO and 3,3′‐diaminobenzidine (DAB) chromogen substrate (Dako). All samples were counterstained with hematoxylin, dehydrated and mounted using the XL High Throughput Autostainer and the Robotic Coverslipper (Leica). The ER (Confirm anti‐ER (SP1), Ventana), PR (Confirm anti‐PR 1E2, Ventana), and HER2 (Confirm anti‐HER2/neu 4B5, Ventana) immunohistochemistry staining for the original patient tissues and PDEs was performed using the Ventana BenchMark Ultra automated slide stainer (Roche) at Mater Pathology (Australia). The ER (Confirm anti‐ER (SP1), Ventana) for PDEs was performed using Ventana BenchMark Ultra automated slide stainer (Roche) at Histology Core, Translational Research Institute, Australia.

### Quantification

IHC staining was quantified using the positive cell detection tool of the QuPath software (v0.2.0‐m9).^[^ ^32^ ^]^ This tool allows the detection of DAB‐positive stained cells in a defined area. All images were set as Brightfield H‐DAB or Brightfield H&E type accordingly. For quantification, the area of analysis was set to the tissue excluding the surface and surrounding hydrogel to only quantify within the explant. The surrounding hydrogel and potential tissue folds were excluded, due to the presence of trapped staining, to eliminate interference from artifact. Additional settings for the positive cell detection were performed using the setting of optical density sum. The percentage of positive cells in each tissue (*n* = 3) was calculated by the QuPath software. Results are presented as the percentage of positive cells relative to the total number of cells across all sections per condition for each donor where the total number of cells was determined by hematoxylin counterstaining.

## Conflict of Interest

The authors declare no conflict of interest.

## Supporting information

Supporting Information

## Data Availability

The data that support the findings of this study are available on request from the corresponding author. The data are not publicly available due to privacy or ethical restrictions.
