# Supplementary material for: Exploring the Potential of PEG‐Heparin Hydrogels to Support Long‐Term Ex Vivo Culture of Patient‐Derived Breast Explant Tissues
Source: Adv Healthc Mater. 2023 Jan 4;12(14):2202202. doi: 10.1002/adhm.202202202 (PMC11469079; doi:10.1002/adhm.202202202)
Supplement: Supplementary file 1 — Supporting Information [file ADHM-12-2202202-s001.pdf]

ADVANCED HEALTHCARE MATERIALS

Supporting Information

for *Adv. Healthcare Mater.*, DOI 10.1002/adhm.202202202

Exploring the Potential of PEG-Heparin Hydrogels to Support Long-Term Ex Vivo Culture of Patient-Derived Breast Explant Tissues

*Maria K. Koch, Akhilandeshwari Ravichandran, Berline Murekatete, Julien Clegg, Mary Teresa Joseph, Madison Hampson, Mitchell Jenkinson, Hannah S. Bauer, Cameron Snell, Cheng Liu, Madeline Gough, Erik W. Thompson, Carsten Werner, Dietmar W. Hutmacher, Larisa M. Haupt and Laura J. Bray**

Supporting Information

Exploring the potential of PEG-Heparin hydrogels to support ex vivo culture of patient-derived breast explant tissues

*Maria K. Koch, Akhilandeshwari Ravichandran, Berline Murekatete, Julien Clegg, Mary Teresa Joseph, Madison Hampson, Mitchell Jenkinson, Hannah Bauer, Cameron Snell, Cheng Liu, Madeline Gough, Erik W. Thompson, Carsten Werner, Dietmar W Hutmacher, Larisa M. Haupt, Laura J Bray**

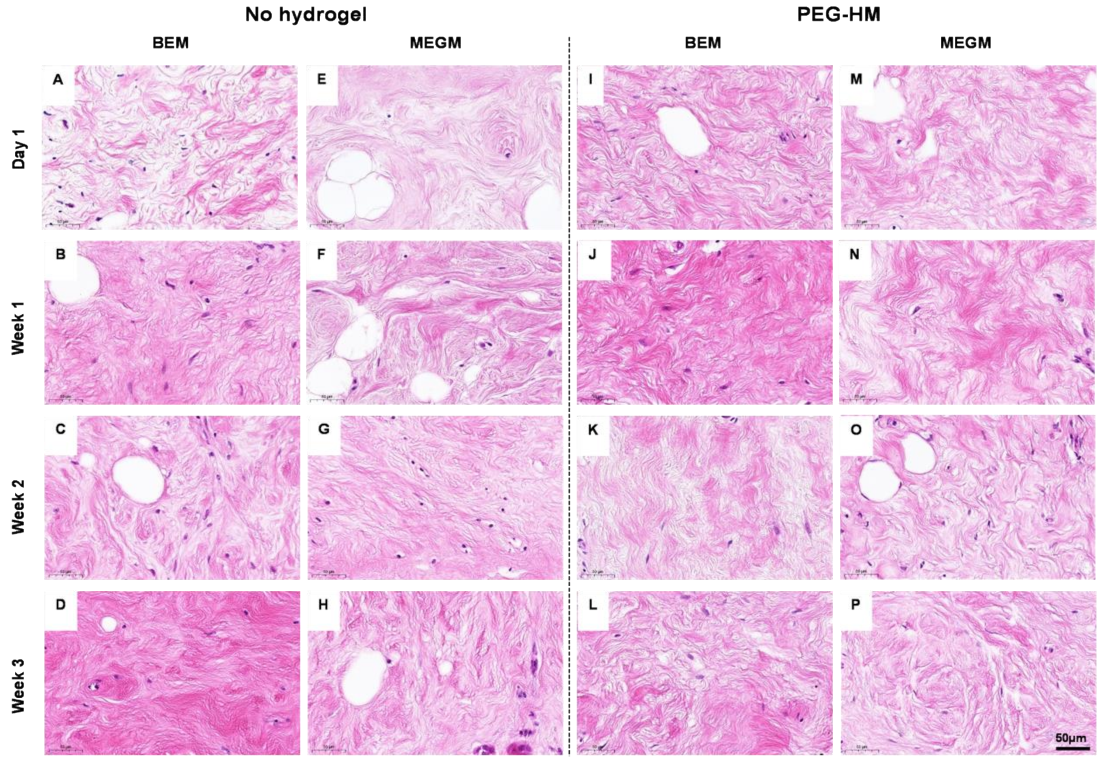

Figure S1. Histology of human mammary stromal tissue of over three weeks of culture Tissue explant cultures of normal breast tissue were cultured embedded in starPEG-heparin (PEG-HM) in BEM or MEGM medium. Control tissues were cultured in parallel without prior embedding. For histological examination, explant cultures were fixed at various time points until 21 days and paraffin sections of 4µm thickness were prepared for H&E staining. Representative brightfield images of stromal tissue are shown of NB-1 explant cultured in BEM medium embedded in hydrogel (i-l), without hydrogel embedding (a-d), as well as cultured in MEGM medium with (m-p) and without (e-h) hydrogel embedding. Scale bar: 50µm.

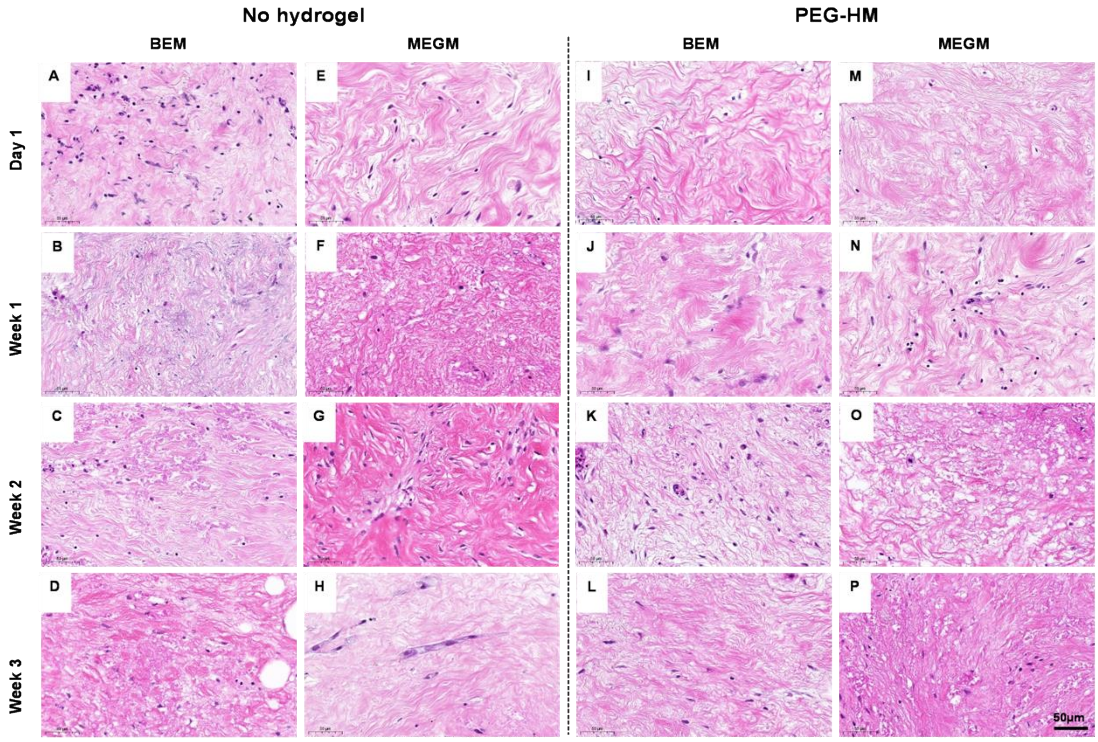

Figure S2. Histology of human breast cancer tissue of over three weeks of culture

Tissue explant cultures of breast cancer tissue were cultured embedded in starPEG-heparin (PEG-HM) in BEM or MEGM medium. Control tissues were cultured in parallel without prior embedding. For histological examination, explant cultures were fixed at various time points until 21 days and paraffin sections of 4µm thickness were prepared for H&E staining. Representative brightfield images of stromal tissue are shown of BC-5 explant cultured in BEM medium embedded in hydrogel (i-l), without hydrogel embedding (a-d), as well as cultured in MEGM medium with (m-p) and without (e-h) hydrogel embedding. Scale bar: 50µm.

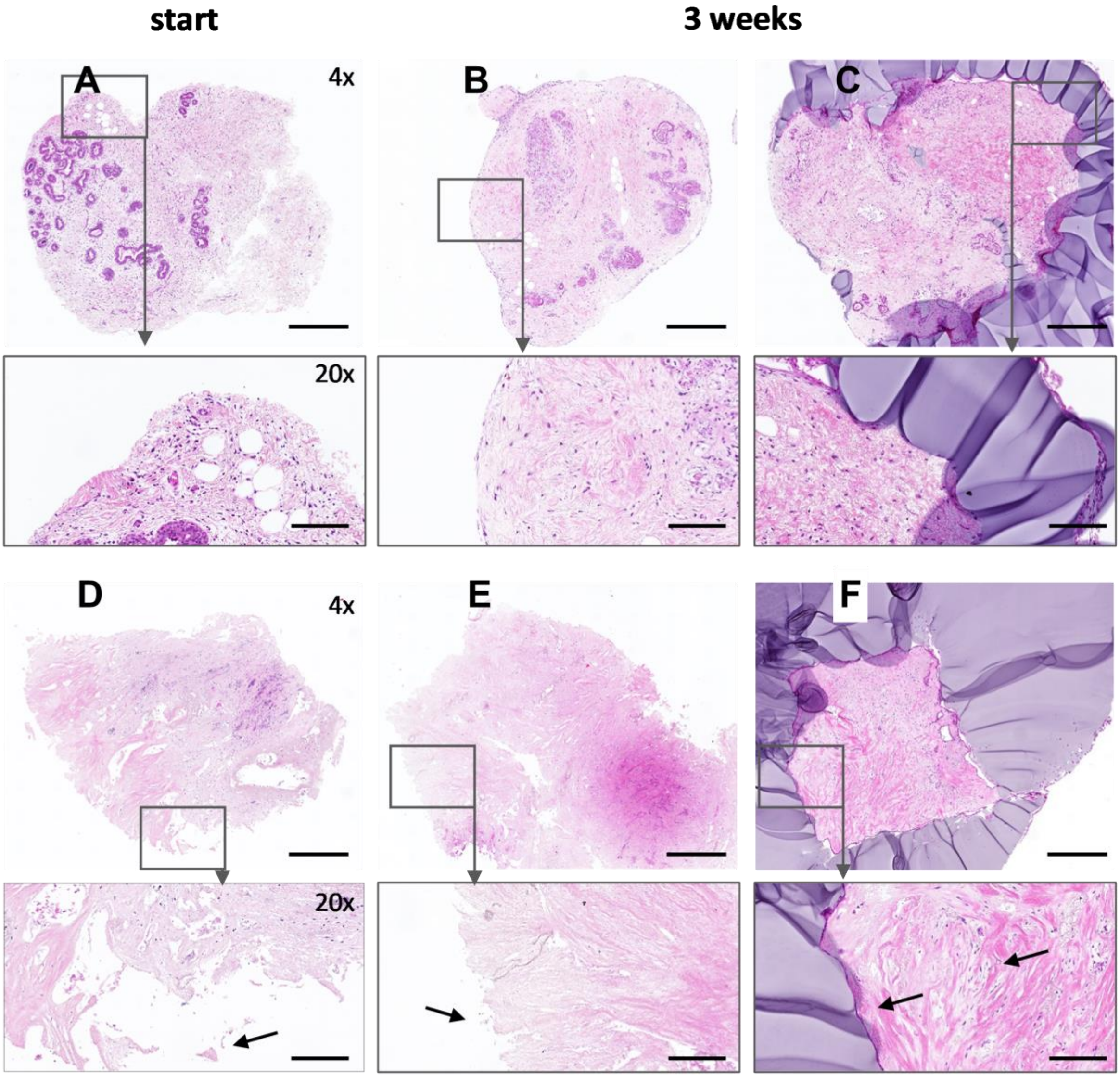

Figure S3. Comparison of tissue surface between culture start and 21 days cultured samples of different donors

Mammary explants were cultured for up to three weeks supported by a surrounding layer of PEG-HM hydrogel. Differences in the appearance of the explant surface over time could be observed after H&E staining of 4µm thick slices and representative brightfield images are displayed from two different donors. The figure shows the complete cross-section of the explant of each donor on the beginning of the culture (A+D) and after three weeks without (B+E) and with hydrogel (C+F). Additionally, a 20x magnified area of the explant surface is shown. The comparison of donor tissue displaying firm tissue structure (A-C) in comparison to donor tissue displaying weaker tissue structure (D-F) showed the supportive effect of PEG-HM hydrogel on less compact tissue. Scale bar 4x: 500µm; 20x: 200µm.

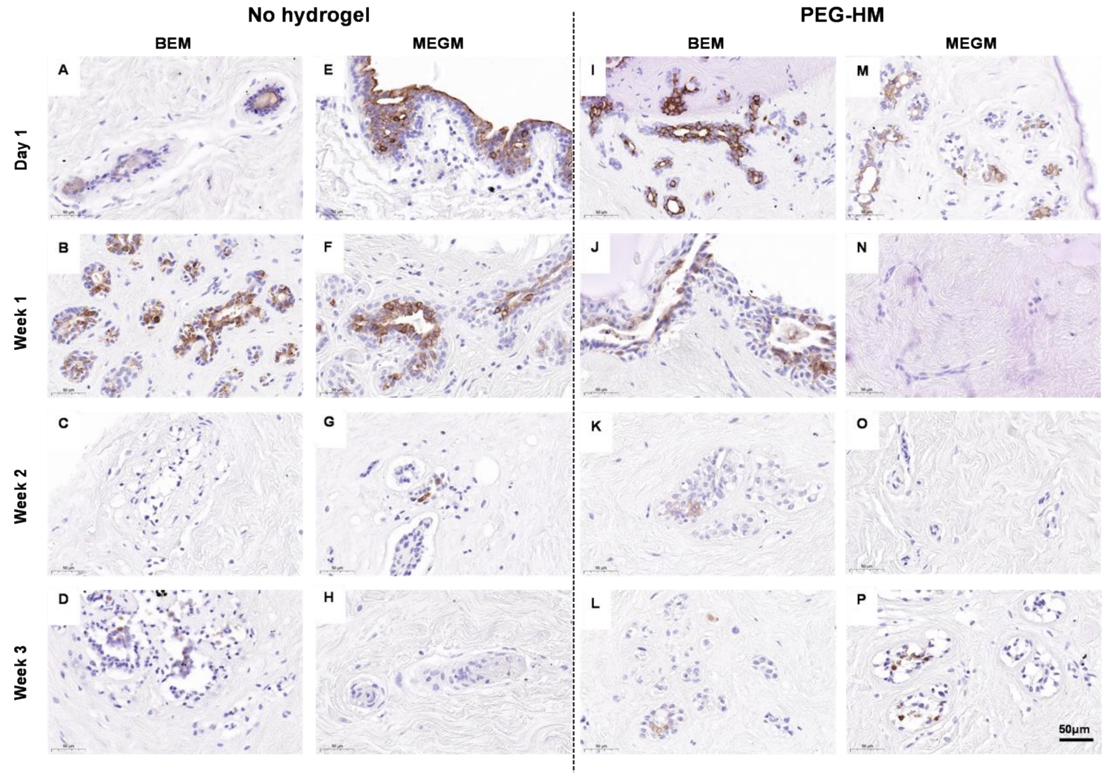

Figure S4. Epithelial staining of normal breast (NB-1) explant cultures

Representative brightfield images of epithelial glandular structures are shown of NB-1 explants stained for Cytokeratin 8/18 cultured in BEM medium without hydrogel embedding (A-D), embedded in hydrogel (I-L), as well as cultured in MEGM medium without hydrogel embedding (E-H) and embedded in hydrogel (M-P). Scale bar: 50µm.

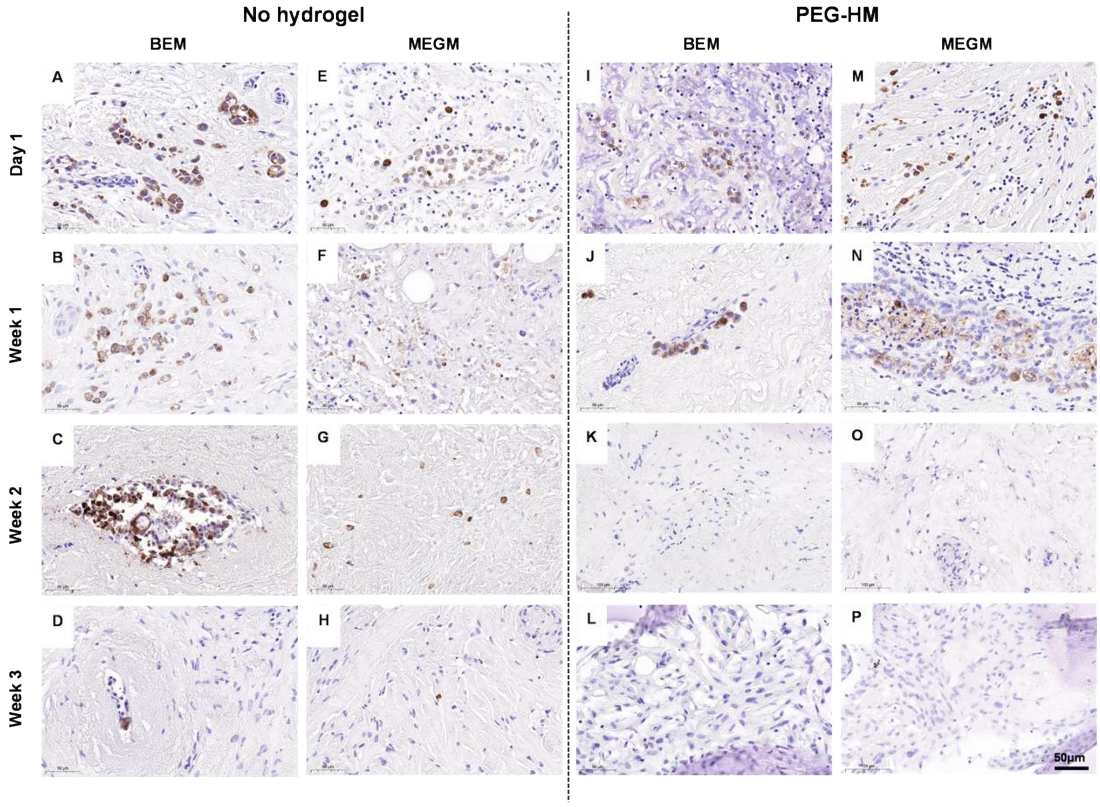

Figure S5. Epithelial staining of breast cancer (BC-5) explant cultures
Representative brightfield images of BC-5 explants stained for Cytokeratin 8/18 cultured in BEM medium without hydrogel embedding (A-D), embedded in hydrogel (I-L), as well as cultured in MEGM medium without hydrogel embedding (E-H) and embedded in hydrogel (M-P). Scale bar: 50μm.

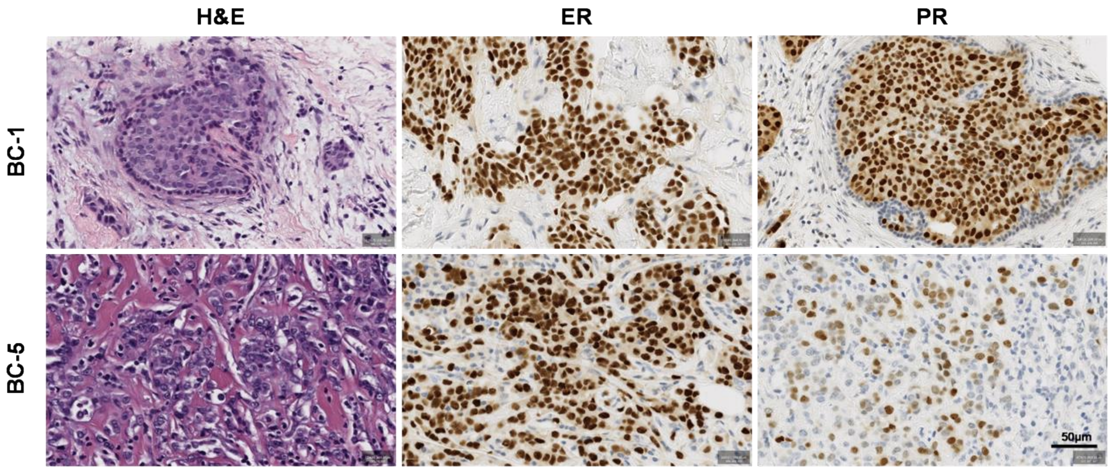

Figure S6. H&E and ER/PR staining for BC-1 and BC-5
Donor tissues obtained in the hospital were stained for testing receptor status. Representative images of H&E, ER, and PR staining for donors BC-1 and BC-5 are presented to function as a baseline for *ex vivo* cultures of the respective donors. Scale bar: 50μm.

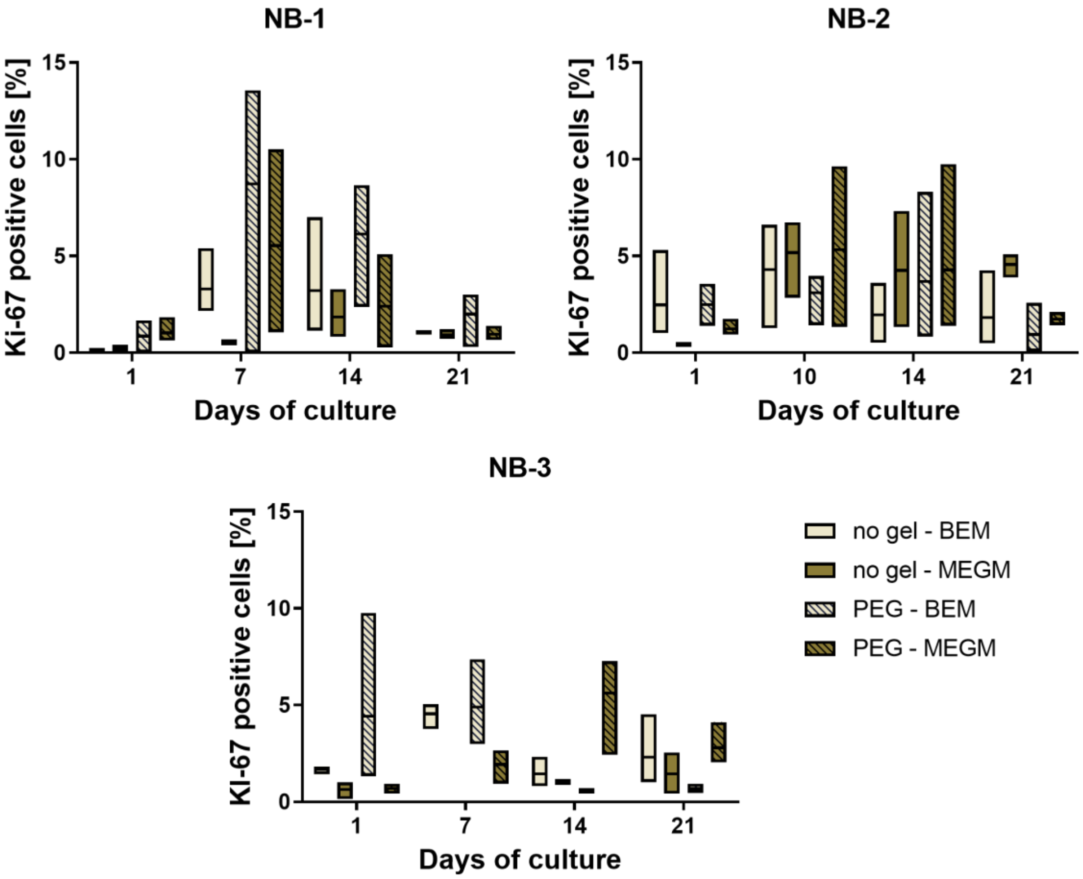

Figure S7. Ki-67 quantification of normal human breast explant cultures
Proliferation in normal human breast tissue explants at various time points and culture conditions was determined immunohistochemically by staining against Ki-67. Positive cells were quantified for the whole explant region of triplicates of each culture condition and differences can be seen between the various time points and culture conditions of NB-1, NB-2, and NB-3 PDEs.

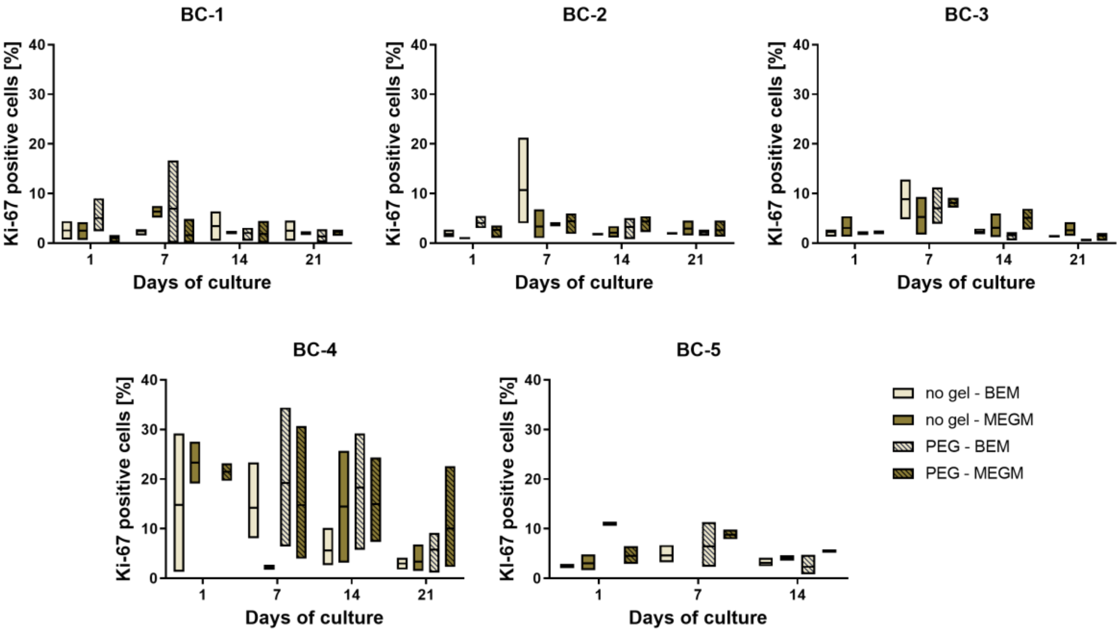

Figure S8. Ki-67 quantification of human breast cancer explant cultures
Proliferation in human breast cancer explants at various time points and culture conditions was determined immunohistochemically by staining against Ki-67. Positive cells were quantified for the whole explant region of triplicates of each culture condition and differences can be seen between the various time points and culture conditions of BC-1, BC-2, BC-3, BC-4, and BC-5 PDEs.
